# Leucopenia in Typhoid Fever

**Published:** 1908-04-11

**Authors:** 


### Medicine.

### LEUCOPENIA IN TYPHOID FEVER.

Whenever a patient is suspected to be suffering from typhoid fever, the first laboratory aid to diagnosis is to take a few drops of blood from the lobule of his ear in a small glass pipette, and send it to a laboratory for examination by Widal's test. The latter, which is a " clumping " reaction, is of the greatest value when it is positive; that is to say, when typhoid bacilli mixed with the serum diluted 200 times clump together within half an hour. When the test is positive to this degree there is practically no doubt that the patient's condition is typhoid fever.

The difficulty is that Widal's test is seldom positive until the patient has been ill at least a week; and very often the reaction is only partial and indeterminate for a longer period even than this; quite commonly the reaction is not completely positive for ten days or a fortnight, and sometimes not even until the third or fourth week.

This being so, it is sometimes a boon to know of other clinical methods that may assist in the diagnosis; and it is too often forgotten that the blood in typhoid fever exhibits two other very constant phenomena, namely:

1. An absence of leucocytosis.
2. An absence of relative increase in the polymorphonuclear cells, or even a relative decrease in this variety of leucocyte with a corresponding relative increase in the small lymphocytes.

We have headed this article " leucopenia," but as a matter of fact " absence of leucocytosis " is a much better term for what really occurs. Some authors have laid stress upon there being an absolute diminution of the number of leucocytes per cubic millimetre of blood in typhoid fever patients; that is to say, a real leucopenia: for clinical purposes, however, while absolute leucopenia is not infrequently observed in typhoid fever, quite as often there are normal numbers of leucocytes, and anything between 3,000 and 9,000 per cubic millimetre is compatible with the uncomplicated disease. The test is of equal value the other way round; that is to say, if there is an actual leucocytosis in a case that is supposed to be typhoid fever, the latter diagnosis is extremely unlikely to be the correct one.

The value of this in practice is constantly showing itself. A female patient, for example, was suffering from an illness of obscure onset, accompanied by moderate but continuous pyrexia, diarrhœa with foulness of the motions, a certain amount of abdominal pain, and no abnormal physical signs in the chest. Her symptoms were such that she was certified as a case of typhoid fever by an official of the Metropolitan Asylums Board. The Widal's test was negative, but this was said to be because it was yet too early in the disease for it to be positive, and it was to be repeated in a few days' time. Meanwhile a leucocyte count was made, and the patient was found to have 25,000 white corpuscles per cubic millimetre of her blood. Such a leucocytosis was clearly incompatible with the diagnosis of typhoid fever, and some deep-seated suppuration was suspected. This was searched for more carefully than before, and it was found in the shape of a double pyosalpinx. The latter was operated upon, and the patient's symptoms were relieved, long before the date upon which Widal's test was to be repeated.

Similar assistance in diagnosis between appendicitis and typhoid fever is also to be obtained. Indeed, the fact that leucocytosis is a phenomenon of great rarity in uncomplicated typhoid fever affords a means of differential diagnosis that is most valuable; it is, unfortunately, too little used in practice. The leucocyte count can help one in a suspicious case long before the Widal's reaction can be expected to be positive.

Not only will the total leucocyte count help one by excluding typhoid fever if there is a leucocytosis, but a certain amount of additional assistance can be obtained by making a differential leucocyte count at the same time. In suppurative and in pneumonic processes there is a relative increase in the polymorphonuclear cells even before the leucocytosis is at all marked. In typhoid fever the polymorphonuclear cells are seldom if ever increased; and in a great many cases they are actually diminished, whilst the small lymphocytes are relatively increased. There are, of course, many other conditions besides typhoid fever in which there is a relative increase in the small lymphocytes, so that this change, taken by itself, is of little significance. Taken in conjunction, however, with clinical symptoms and pyrexia, which already suggest the possibility of typhoid fever, and also in conjunction with the fact that there is no leucocytosis, the relative lymphocytosis is an additional ground for believing the diagnosis of typhoid fever to be correct. Conversely, a similar patient whose differential leucocyte count shows a considerable increase in the polymorphonuclear cells is much more likely to have a deep-seated abscess or pneumonia than enteric fever. The following are typical of three differential leucocyte counts, for normal blood, for typhoid fever, and for suppuration or for pneumonia respectively:

| | Normal Blood. % | Typhoid Fever. % | Pus or Pneumonia. % |
|---|---|---|---|
| Small lymphocytes ... | 25 | 32 | 18 |
| Large lymphocytes ... | 8 | 8 | 6 |
| Polymorphonuclear cells... | 65 | 58 | 74 |
| Coarsely granular eosinophile cells | 2 | 2 | 2 |

The absence of leucocytosis and the absence of relative increase in the polymorphonuclear cells are indications which hold good so long as the typhoid fever is not complicated by perforation or by the formation of pus—an empyema of the gall-bladder, for example. Their main diagnostic advantage is in the earlier stages of the disease. Later on the Widal test supplies all that can be desired; but during the first ten days of the illness, when Widal's reaction is not yet positive, or at most only partial and doubtful, much assistance in the diagnosis of typhoid fever can be obtained by making both a total and a differential leucocyte count.

